# Differences in the Early Development of Human and Mouse Embryonic Stem Cells

**DOI:** 10.1371/journal.pone.0140803

**Published:** 2015-10-16

**Authors:** R. Gabdoulline, W. Kaisers, A. Gaspar, K. Meganathan, M. X. Doss, S. Jagtap, J. Hescheler, A. Sachinidis, H. Schwender

**Affiliations:** 1 Center for Bioinformatics and Biostatistics, Biological Medical Research Center, Heinrich Heine University, Universitätsstrasse 1, 40225 Düsseldorf, Germany; 2 Center of Physiology and Pathophysiology, Institute of Neurophysiology and Center for Molecular Medicine Cologne (CMMC), University of Cologne, Robert-Koch-Str. 39, 50931 Cologne, Germany; 3 Mathematical Institute, Heinrich Heine University, Universitätsstrasse 1, 40225 Düsseldorf, Germany; The University of Hong Kong, HONG KONG

## Abstract

We performed a systematic analysis of gene expression features in early (10–21 days) development of human *vs* mouse embryonic cells (hESCs *vs* mESCs). Many development features were found to be conserved, and a majority of differentially regulated genes have similar expression change in both organisms. The similarity is especially evident, when gene expression profiles are clustered together and properties of clustered groups of genes are compared. First 10 days of mESC development match the features of hESC development within 21 days, in accordance with the differences in population doubling time in human and mouse ESCs. At the same time, several important differences are seen. There is a clear difference in initial expression change of transcription factors and stimulus responsive genes, which may be caused by the difference in experimental procedures. However, we also found that some biological processes develop differently; this can clearly be shown, for example, for neuron and sensory organ development. Some groups of genes show peaks of the expression levels during the development and these peaks cannot be claimed to happen at the same time points in the two organisms, as well as for the same groups of (orthologous) genes. We also detected a larger number of upregulated genes during development of mESCs as compared to hESCs. The differences were quantified by comparing promoters of related genes. Most of gene groups behave similarly and have similar transcription factor (TF) binding sites on their promoters. A few groups of genes have similar promoters, but are expressed differently in two species. Interestingly, there are groups of genes expressed similarly, although they have different promoters, which can be shown by comparing their TF binding sites. Namely, a large group of similarly expressed cell cycle-related genes is found to have discrepant TF binding properties in mouse *vs* human.

## Introduction

Comparing gene expression properties of human and mouse embryonic stem cells (hESCs and mESCs, respectively) gives an invaluable insight into evolutionary conserved features of ESCs. Several markers that distinguish undifferentiated ESCs from their differentiated progeny could be identified [1]. Hundreds of genes were found to be differentially expressed in undifferentiated hESCs compared with their differentiated counterparts, and this list intersects with published mouse embryonic stem cell data, pointing to the existence of a "core molecular program" possibly including ligand/receptor pairs and secreted inhibitors of the FGF, TGFbeta/BMP, and Wnt pathway [2]. Using various methods, evolutionarily conserved and divergent transcriptional co-expression relationships regulating pluripotency were identified [3–5]. Conserved mechanisms of transcriptional regulation was found by analyses of sequences both aligned and non-aligned between different genomes with a probabilistic segmentation model to systematically predict short DNA motifs that regulate gene expression [6]. Besides the core Oct4-Sox2-Nanog circuitry, accumulating regulators including transcription factors, epigenetic modifiers, microRNA, and signaling molecules have also been found to play important roles in preserving pluripotency [7].

At the same time important differences were found between human and mouse ES cells. Comparing gene expression patterns of mouse and human ES cells by immunocytochemistry, RT-PCR, and membrane-based focused cDNA array analysis showed that significant differences exist in expression of vimentin, beta-III tubulin, alpha-fetoprotein, eomesodermin, HEB, ARNT, and FoxD3 as well as in the expression of the LIF receptor complex LIFR/IL6ST (gp130) [1]. Profound differences in cell cycle regulation, control of apoptosis, and cytokine expression were observed. Importantly, the patterns of gene expression observed in H1 cells were similar to that of two other human ES cell lines tested (line I-6 and clonal line-H9.2) and to feeder-free subclones of H1, H7, and H9, indicating that the observed differences between human and mouse ES cells were species-specific [1]. (In original publication the term “profile” is used to describe the overall state of gene expression. In this paper, we often use the term “profile” for the shape of time-series expression levels, therefore other than “profile”, for example, the term “pattern” will be used for other gene expression features.) Growth factor requirements for hESC and mESC maintenance are different, with LIF required only for mESCs. Transcription factor FoxD3 and STAT3 expression is essential only in mESCs and dispensable in hESCs. Analysis of co-expression cross-species clustering (SCSC) approach [8] together with protein-DNA binding data indicated that the KLF2/4/5 transcription factors, although critical to maintaining the pluripotent phenotype in mouse ES cells, were decoupled from the OCT4/SOX2/NANOG regulatory module in human ES cells. Two of the target genes of murine KLF2/4/5, LIN28 and NODAL, were rewired to be targets of OCT4/SOX2/NANOG in human ES cells. Moreover, there are signal transduction components that were induced in pluripotent ES cells in either a conserved or a species-specific manner. The study of transcriptome and epigenome of mouse and human pluripotent stem cells also show critical differences in gene expression of specific pathways as well as in bivalent modification of promoters by H3K4 and H3K27 trimethylation [9].

In this work, we compare the development process of mESCs and hESCs. To do that, expression profiles of orthologous genes, derived from time-course microarrays [10, 11] are used. Differences in the process of development should be expected to be large. For example, comparison of preimplantation embryonic development of three mammalian species (human, mouse, bovine) [12] showed significant differences in inferred gene regulatory networks. The differences should be awaited also because of using the data from 3’ expression arrays and comparing the signals of probes from different platforms for human and mouse, which are hardly comparable directly. However, recent studies showed that expression levels of orthologous genes are quite comparable, when, for example, High-Density Exon Arrays are used [13]. Therefore, one might still expect the expression levels of genes in human and mouse to be similar or at least comparable after additional processing of microarray data. In our study, we show that renormalized time-course profiles of gene expression of orthologous genes in humans and mice are highly correlated for majority of genes, which makes quantification of differences and similarities meaningful. Similarities are even more evident, when the expression profiles are clustered and the average profiles of clusters are compared.

We quantify similarities and differences for coexpressed groups (23 groups here) of genes, as well as for enrichment of GO biological processes [14]. Further, we compare these clusters both using sequence alignment of the promoters of their genes as well as enumerating transcription factor binding sites on the promoters, and identify gene groups that possibly have different expression features due to different transcriptional regulation.

## Results

### Organ development differences

**Fig 1:**
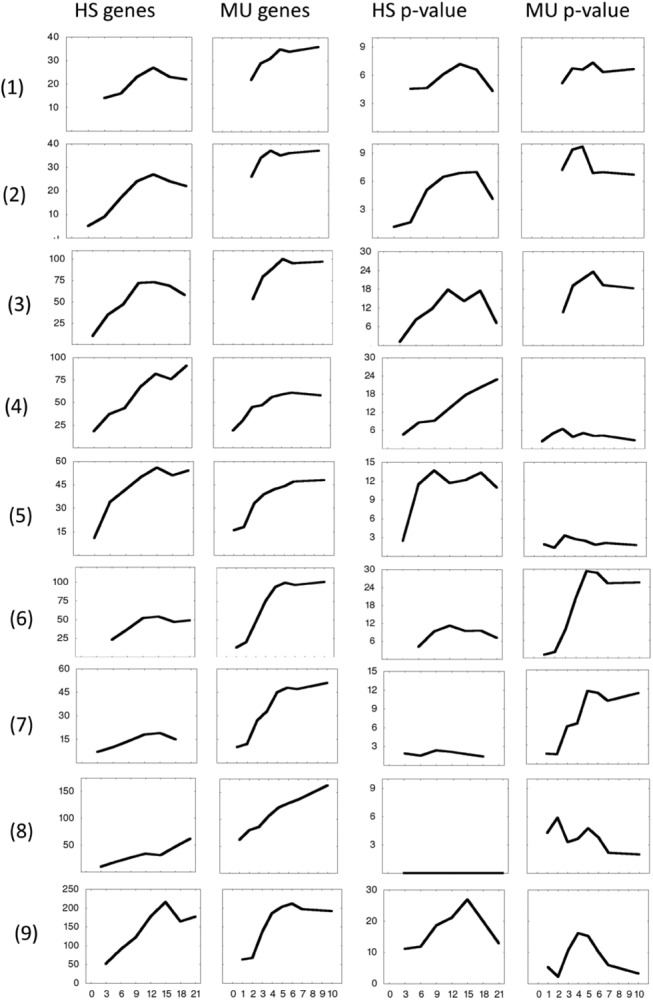
Number of upregulated (compared to day 0) genes and enrichment p-values for several organ development categories and TFs. Gene numbers (left two columns) and enrichment p-values (-log_10_(p), right two columns) for up-regulated genes of GO Biological Processes: (1) kidney development, (2) lung development, (3) skeletal system development, (4) neuron development, (5) sensory organ development, (6) vascular development, (7) muscle tissue development. Notation MU stands for mESC and HS for hESC data. (8) Down- and (9) upregulated genes, annotated as transcription factors.

We compare early development of human *vs* mouse ESCs (hESCs *vs* mESCs). hESCs are quantified by gene expression microarrays at development days 0, 3, 6, 9, 12, 15, 18 and 21, with 0 corresponding to undifferentiated state. mESCs gene expression is measured on days 0, 1, 2, 3, 4, 5, 6, 7 and 10. Every time point is represented by 3 replicates. At each development time point, we extract genes that are upregulated with respect to undifferentiated state and classified as relevant to specific organ development GO Biological Process. The number of genes and enrichment p-values are calculated and presented in Fig 1. In certain cases the p-values give another picture than the number of genes because of differences in the total number of classified genes in human and mouse.

10-day kidney/lung/skeletal system development of mESCs compare quite well to 21-day development of hESCs. Neuron and sensory organ development involve larger number of genes in hESCs, while vasculature development is more enhanced in mESCs. The largest differences are seen for muscle tissue development related genes: In hESCs, they are practically not up-regulated during 21 days of development.

Comparison of different development time intervals (10-day for mESCs *vs* 21-day for hESCs) appeared to be appropriate throughout our analysis; this may be caused by analogous difference in population doubling time (30–35 h for hESCs *vs* 12–15 h for mESCs [15]).

### Cluster analysis of expression profiles

There is no direct correspondence between the probe sets in arrays HG-U133_Plus_2 (human) and mouse4302 (mouse) considered in this paper. However, probe sets can be mapped to genes, for which the correspondence can be established, as between orthologous genes, for example, from Mouse Genome Informatics database (http://www.informatics.jax.org/). For each gene, we select one probe set, which has the largest change in expression level. In this way, a probe set from human array is unambiguously related to a probe set from mouse array. Further, expression profiles are normalized, in order to compare the shapes of expression profiles, rather than the absolute values of signals.

**Fig 2:**
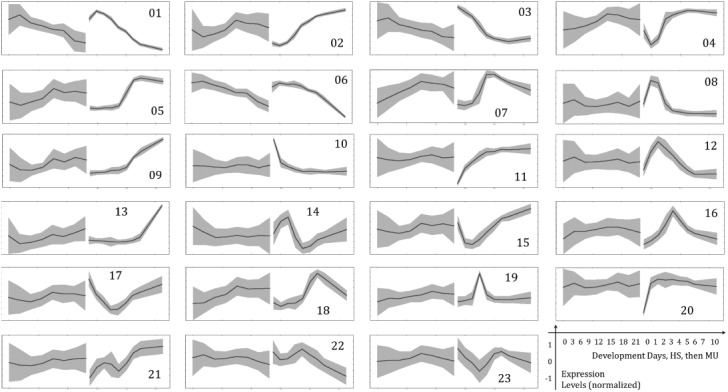
Mouse clusters. 23 clustered expression profile sets are shown. The right part of each graph is the expression profile of (clustered) mouse genes and the left–for corresponding human genes. A line on the graphs is an average expression and grey areas indicate standard deviation of individual expression values with respect to average. Before clustering the standard deviation of expression profiles was set to 1, in order to make the results independent of specific probe set properties.

**Fig 3:**
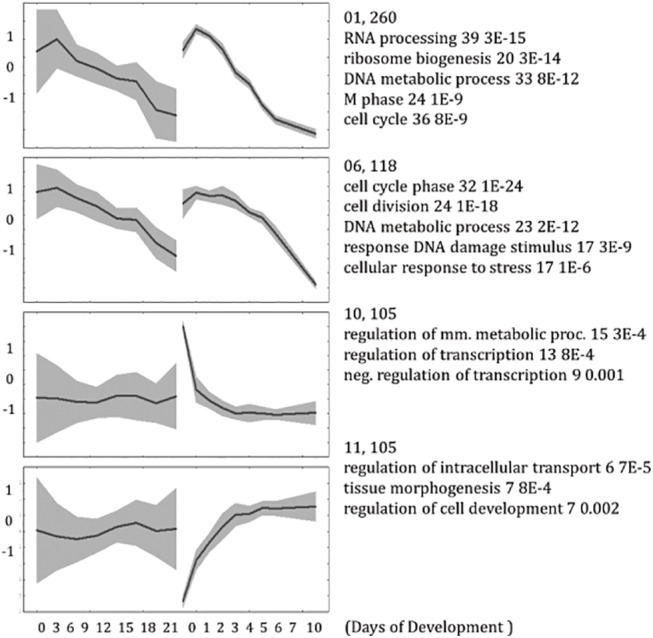
Selected clusters, annotated with GO terms. mESC profiles are clustered and shown on the right part of the graphs; respective hESC gene profiles are on the left. First 2 examples are for the most similar time-series expression, the other 2 –for the most dissimilar cases. This is an excerpt from S7 Table.

Various comparisons were performed. First, correlation of profiles in each orthologous gene pair is calculated. In the second approach, we cluster expression profiles in one case and then look at the expression profiles of orthologous genes of the other case (i.e. human and then mouse, or mouse and then human). Different clustering methods were tried as well to ensure that the conclusions do not depend on selected clustering method. Detailed descriptions of the calculated clusters are available in the supporting information. Gross pictures of similarities and dissimilarities are shown in the Fig 2. Some remarkable similarities and dissimilarities are presented in Fig 3.

The most conserved feature is likely the down-regulation of cell-cycle related genes in both human and mouse. There are about 300 of these genes, which expression drops after a short delay. The most dissimilar behavior is seen for the list of about 100 genes, enriched by regulators of transcription, which are down-regulated in mouse within the first days, but show no apparent differential regulation in human, implying that detectable differences exist in transcriptional regulation in early phase of development.

Some expression profiles show peaks at different stages of development, which are rarely conserved. For example, neuron development related genes are upregulated peak-wise at the 2^nd^ day in mouse (see S7 Table), while in human the up-regulation continues till the last, i.e. the 21^st^ day, of measurement. Embryonic morphogenesis related genes have a peak of up-regulation at 3-4^th^ days in mouse, gene expression of corresponding genes in human also show peaks, but not exactly at the respective time points. Therefore, conservation of peaks cannot be established. One of the difficulties for assigning the correspondence is that we compared 0–10 days of mESCs development to 0–21 day development of hESCs, i.e. compared timescales in human and mouse development are different, although this scaling was found to be the best (see paragraph “One-to-one analysis of expression profiles” below). It can be shown that the peaks are not due to the (low) quality of particular chips, because in most of cases they are found in all 3 independently measured replicates.

**Fig 4:**
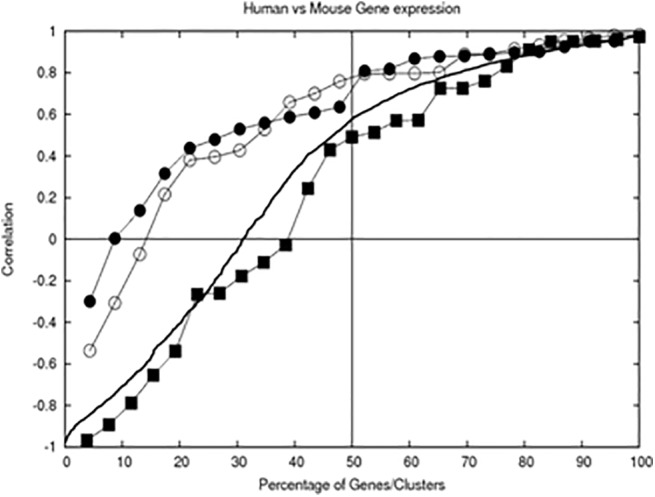
Correlation of profiles of gene groups/clusters and individual gene pairs, sorted and drawn as a function of percentage of total number of group/gene pairs. The bold line shows a correlation of expression profiles of the same gene in human vs in mouse. Correlation of average profiles of gene groups are also shown: line with filled circles -clustered using mESC gene expression profiles, line with open circles—clustered using hESC profiles, line with filled squares—co-clustered. The correlations were sorted and drawn as a function of percentage of all group/gene pairs, allowing comparison of cases with different number of pairs. Significance of correlation p<0.05 is at correlation coefficient larger than 0.67 for 9-point expression profiles and 0.71 for 8-point profiles.

Visual inspection shows that there are high correlations in expression profiles of clustered genes. When quantified by calculating Pearson correlation coefficients, positive correlations can be found for about 90 percent of clusters, see Fig 4. In the same figure, we show the correlation of profiles of individual gene pairs, revealing that about 70% of gene expression profiles are positively correlated. We also performed additional clustering using all expression data points (both for human and mouse) and compared average profiles of separated out human-gene-related time points *vs* mouse-gene-related time points. The results from this analysis are referred to as “co-clustering” and show similar features as individual gene pairs.

### Degree of gene expression differences

Not all orthologous genes are expressed in both cells. To estimate a degree of diversity, we analyzed Presence/Absence calls from 24 human and 27 mouse arrays for each gene by mapping calls for probe sets to a gene as a maximum presence call for all relevant probe sets corresponding to this gene. The results are summarized in Table 1. For all established 15073 orthologous gene pairs, it appears that about 15% can be considered to be not expressed in both ESCs. Further, another 15% is not expressed in hESCs, but expressed in mESCs. Twice less is not expressed in mESCs, but expressed in hESCs. 60% of genes were found to be expressed in both cells. The same analysis was performed for transcription factors and the same diversity was found. For this analysis we used the list of transcription factors from hierarchical classification of human transcription factors [16]. The total number of transcription factors mapped to both cell gene lists was 1072.

**Table 1:** Percentages of genes and transcription factors found to be expressed or not expressed in hESCs and mESCs.

Presence calls	<3	<4	<5	<3	<4	<5
	% of 1072 TFs			% of 15073 genes		
hESC- mESC-	11.94	13.90	15.21	12.70	14.43	15.63
hESC- mESC+	16.88	17.07	16.51	15.39	15.46	15.67
hESC+ mESC-	7.28	7.46	7.46	7.13	7.39	7.48
hESC+ mESC+	63.90	61.57	60.82	64.77	62.71	61.22

Percentage of orthologous genes and transcriptions factors for different combination of their expression status (+ for expressed and–for not expressed) in human (hESC) and mouse (mESC) cells.

Even larger differences in gene expression features are seen if upregulated genes assigned to development related GO Biological Process categories or transcription factor activity are counted. The number of common and different genes responsible for the same process is shown in Table 2.

**Table 2:** The number of genes and the overlap of lists of human and mouse genes, FC 2 upregulated at least on one time point, and assigned to indicated GO category.

GO	Description	hESC		mESC		common	
		#	%	#	%	#	%
GO:0001501	skeletal system development	92	29.0	130	47.4	41	18.0
GO:0001822	kidney development	39	40.6	50	49.0	19	23.8
GO:0001944	vasculature development	68	27.2	135	54.9	42	20.4
GO:0007423	sensory organ development	76	33.6	85	35.4	33	16.4
GO:0030324	lung development	37	38.5	51	46.4	20	22.5
GO:0048666	neuron development	117	34.5	110	37.8	49	20.2
GO:0060537	muscle tissue development	25	20.0	68	50.7	15	14.2
GO:0003700	transcription factor activity	209	23.1	239	33.2	79	13.1

Percentage of genes is to total number of genes in respective GO category, which may be different in the two organisms. Percentage for common genes is to the number of common orthologous genes in the GO category.

The overlaps between mouse and human gene lists are overrepresentations, although only 19% (on average over the 8 categories) of common genes are upregulated in both cases. This should be compared to 31 and 44% in hESCs and mESCs. On average over all categories, there are more upregulated genes in mESCs than in hESCs (t-test based p-value 0.0014). In each category, the fraction of upregulated genes is larger in mESCs, than in hESCs.

### One-to-one analysis of expression profiles

We also compared expression profiles of 2587 differentially regulated in both mESCs and hESCs by calculating Pearson correlation coefficients. Comparisons can be done different ways, since we have different time-series measurements for different species, namely, days 0–10 for mESCs and days 0–21 for hESCs. One can select different time intervals over which the profiles are compared. We compared 10 hours of mouse ESC development to varying time intervals of human ESC development, changing this interval from 12 to 21. Comparing 10 to 21 hours gave the largest number of positively correlated genes (68.3% *vs* 64.2% while comparing 10 to 12), as well as the number of genes, with correlation higher than 0.693, statistically significant at p-value 0.05 at 8.5 degrees of freedom (39.1% *vs* 34,8 while comparing 10 to 12), and therefore, was accepted as the most appropriate. We also performed Principal Component Analysis (PCA) of overall gene expression profiles, and found higher similarity between mouse 10-hour and human 21-hour.developments, see Table 3. This conclusion is supported by observed differences in population doubling time 12–15 h for mESCs *vs* 30–35 h for hESCs [15], also implying that early hESC development process is roughly 2 times slower than mESC development.

**Table 3:** Correlation (quantified by Pearson correlation coefficient) of eigenvectors from Principal Component Analysis (PCA) at different time intervals of hESC development with eigenvectors of 0–10 hour mESC development.

Compared eigenvectors		Overlapped hESC development intervals			
HS	MU	0–12 hours	0–15 hours	0–18 hours	0–21 hours
1	1	0,936	0,936	0,983	**0,99**
1	2	0,984	0,992	**0,994**	0,993
2	2	0,819	0,827	0,787	**0,84**
3	3	0,792	0,792	0,795	**0,838**
4	4	0,833	0,799	**0,897**	**0,897**
5	5	0,265	0,455	**0,815**	0,547
6	6	0,637	0,707	**0,909**	0,577
7	7	0,165	0,167	**0,562**	0,275

For correlating eigenvectors we interpolated expression values in both cases to 11 equidistant points, concatenated 2 sets of profiles and performed PCA. Then the 22-point eigenvectors were split onto 2 parts, corresponding to mESC and hESC points, and compared. The first 2 eigenvectors were semi quantitatively similar; therefore eigenvector 1 was also compared to eigenvector 2.

The results are shown in Fig 4 (as a line) and S3 Table.

### Analysis of promoters

Expression of genes is expected to be regulated by transcription factors acting on promoters of these genes. Identifying these transcription factors may shed light on the reasons for similarities and differences in gene expression features. In order to identify the relevant transcription factors, we search promoters of clustered genes for the presence of TFBS (transcription factor binding sites). For a given cluster, both human and mouse promoters were analyzed, and the lists of discovered TFBSs were compared.

We used a collection of TFBSs taken from TRANSFAC [17], UNIPROBE [18] and JASPAR [19]. TFBSs were tested for the presence on the promoters of clustered genes and compared to specially prepared “background” set of promoters of non-regulated (though expressed, according to their Presence calls) 500 genes. Over-representation is quantified by p-values, calculated from hypergeometric probability distribution for obtaining observed density of binding sites in a given set of promoters, when the density of binding sites in promoters of “background” set is assumed to be expected [20, 21].

**Fig 5:**
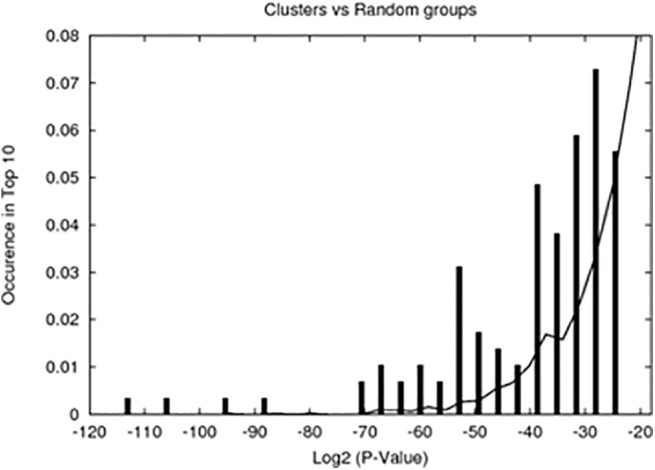
Histogram of p-values (-log2 scale) calculated for TFBSs of clustered gene groups *vs* random groups of genes of the same size. TFBS overrepresentation p-values for clustered gene groups are shown as impulses, for random groups—as lines. According to this comparison, TFBSs assigned to the group with overrepresentation p-value less than 10^−9^ (log_2_P<-29.9) can be considered to be less likely to appear by a chance.

Groups of genes derived by clustering are assigned TFBSs, whose presence on the promoters has low p-values. In addition to calculating these p-values, indicating significance of single TFBSs for regulation of these gene groups, we performed a separate test to check if our clustered gene groups show TFBS enrichment better than random groups of genes of the same size. These checks are necessary because of the multiple testing nature of TFBS assignments, when even randomly chosen groups can have certain TFBSs assigned a low p-value. The results are shown in Fig 5. Indeed, our groups have assigned TFBSs with far smaller p-values than the random groups, i.e. within this approach we cannot only examine general similarity of the promoters in terms of TFBSs, but also assign TFBSs, which may be relevant to the regulation of particular groups.

A cutoff for p-values at 10^−9^, derived from comparison to random groups, is too small, in a sense that not every group has the binding site with this degree of significance. Therefore, for comparing promoters, we used another cutoff, namely 7.6 10^−5^, which corresponds to 0.05 divided by a total number of different TFBSs used (i.e. to a Bonferroni correction of the cutoff), and with which every group has at least 5 significantly overrepresented TFBSs. Overlap of TFBS lists was also quantified by another p-value, calculated from hypergeometric probability distribution for obtaining observed number of overlapping TFBSs between 2 lists of known size from total number of TFBSs. A low p-value means that the lists of TFBSs are similar. The similarity of TFBS lists implies the similarity of promoters.

**Fig 6:**
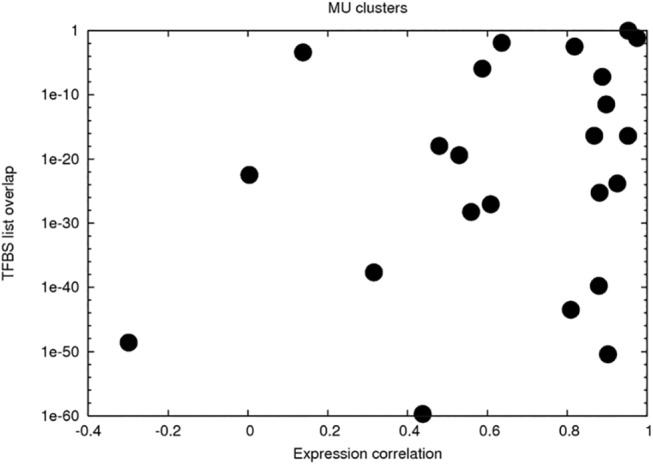
Relation between expression and promoter similarities. The x-axis is for Pearson correlation coefficients of average gene expression profiles of human *vs* mouse gene group pairs. The y-axis is for p-value of overlap of their TFBS-s lists.

We see almost all possible scenarios, indicating that the correlation of gene expression profiles is not directly related to the similarities or dissimilarities of promoter sequences, quantified by the list of TFBSs on them. In majority of cases, the similarity in expression is accompanied by the similarity of promoters (right-bottom of Fig 6). It should be kept in mind, however, that the similarity of the promoters is not sufficient to ensure similar expression. Additional requirement is that the activity of relevant transcription factors is similar in mESCs and hESCs. Thus in majority of cases both requirements are met. There are few cases when hESCs and mESCs promoters have apparently the same TFBSs, but their expression profiles are not correlated, and even anti-correlated (left-bottom in Fig 6). These are likely the cases when differences in the activity of relevant transcription factors can be expected.

Surprisingly, there are cases (clusters) in which the expression profiles in hESCs and mESCs are similar, but TFBSs derived from inspecting relevant promoters are different. This could be due to the incompleteness of our list of TFBSs. However, in one deliberately investigated case, we have seen that hESCs and mESCs promoter sets are indeed different in in the sense of the presence of TFBSs. This is the cluster 1 in the Table 4, one of the two clusters, enriched by cell cycle related genes down-regulated during the development of cells. Sequence alignment using *lalign* [22] does not show significant differences in the sequences of promoters on average, as this can be expected for groups of orthologous genes. However, hESC promoters have motifs, discovered with MEME [23], other than mESC promoters for the same group of genes. Calculated TFBS lists are apparently different for the two sets of promoters (human and mouse), and p-values of TFBS lists used to compare promoters are larger than the previously identified cutoff of 10^−9^. Therefore, our calculated TFBS enrichment of promoters of these genes does not fully explain the properties of their transcriptional regulation.

**Table 4:** Expression and promoter TFBS properties of mESC clusters.

Cluster #	# Genes	PCC of profiles	TFBS list overlap	Top GO Biological Process	# TFBSs with P-value < 10^−9^
1	260	0.953	1.00	RNA processing / Cell cycle	0
2	221	0.879	1.71E-40	Cell proliferation / Skeletal system dev	3
3	183	0.888	6.52E-08	Regulation of transcription	0
4	174	0.902	3.77E-51	Positive regulation cell differentiation	16
5	150	0.952	4.11E-17	Vasculature development	2
6	118	0.975	0.072	Cell cycle phase	0
7	115	0.868	4.28E-17	Heart development	0
8	111	0.608	8.99E-28	Neuron development	4
9	109	0.881	5.65E-26	Response to organic substance	10
10	105	0.138	4.15E-04	Regulation of transcription	0
11	105	0.438	1.95E-60	Regulation intracellular transport	29
12	89	0.316	2.29E-38	Neuron development	22
13	81	0.809	3.28E-44	Regulation of cell proliferation	8
14	72	0.559	5.58E-29	Anti-apoptosis	5
15	70	0.926	1.53E-24	Response to organic substance	13
16	69	0.479	1.11E-18	Embryonic limb morphogenesis	9
17	65	0.587	1.22E-06	Lung development	0
18	65	0.898	3.32E-12	Blood vessel development	0
19	61	-0.299	2.50E-49	Embryonic morphogenesis	16
20	60	0.004	3.38E-23	Sterol biosynthetic process	1
21	50	0.818	0.0034	Regulation skeletal tissue development	1
22	48	0.636	0.013	M phase	0
23	36	0.529	4.07E-20	Response abiotic stimilus	5

For every cluster (1–23) the following properties are listed: The number of genes in cluster; Correlation of expression profiles; p-value of overlap of calculated TFBS lists of human and mouse gene promoters; Top GO Biological Process; The number of TFBSs having overrepresentation p-value < 10^−9^.

Detailed description of gene groups with respect to TFBS lists is shown in Table 4. In the majority of cases both expression profiles and relevant TFBSs are similar, indicating conserved transcriptional regulation. In the cluster 10 there is a weak correlation of expression profiles and a weak overlap of TFBS lists. These are most likely cases in which the genes in human and mouse are differently regulated, as their promoters have different TF binding properties. In clusters 11, 12, 20, and in particular, 19, the profiles are not similar, while TFBS lists are almost the same. This may indicate that involved TFs are either differently expressed or activated in hESCs or mESCs. In clusters 1, 6, the profiles are similar, while no similar TFBS lists are seen. Apparently in these cases, there is no common transcriptional regulation caused by the same TFBSs. More detailed analysis shows that these are the cases, when (1) no significantly overrepresented TFBSs are found at all, and (2) gene expression is a simple down-regulation. Therefore, the viable hypotheses would be that either regulation here is not related to TF binding, or the expression profiles are so simple that clustering approach was not able to identify co-regulated gene group.

**Fig 7:**
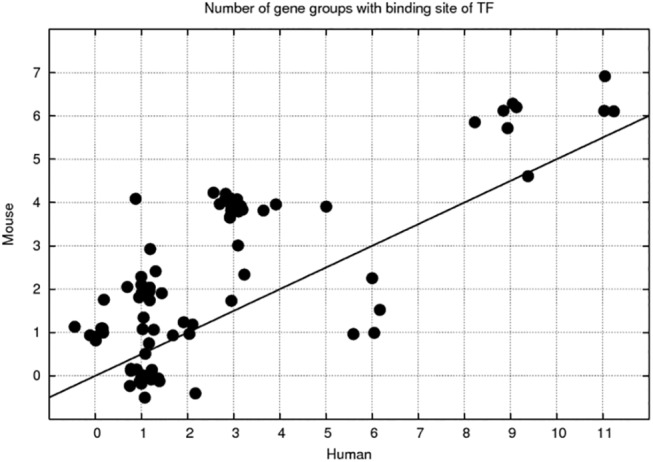
Number of gene groups with binding site of TF. Correlation between the number of gene groups/clusters, having statistically significant overrepresentation of binding sites of 81 TFs, calculated for human and mouse clusters. The points are randomly disposed. The line y = 2 x is drawn for a reference.

Alone the ability of clustering approach to detect co-regulation is out of suspect, since using clustering we find many clusters with clearly assigned TFBSs (Table 3, last column). The lists of all TFs are given in S10 Table; altogether there are 81 TFs that can be called to be responsible for regulation of the development process. S10 Table also shows the number of gene groups (clusters), which are found to be regulated by given TFs. Some TFs appear to regulate many gene groups, for example, Sp1-4, Znf219, Patz1 (POZ-, AT hook-, and zinc finger-containing protein 1), EGR* (Early growth factors), Fox* (Forkhead box proteins), Wt1 (Wilms tumor protein), Tfdp1 (Transcription factor Dp-1), Tfap2 (Transcription factor AP-2 alpha), E2F*, Nrf1 (Nuclear respiratory factor 1), Znf148, Vdr (calcitriol receptor) can be associated with more than 5 clusters (either in mouse or human). Interestingly, the number of clusters, which TFs can regulate, is highly correlated between human and mouse (Pearson correlation coefficient 0.75, see Fig 7), probably showing that ubiquitous TFs are the same in human and mouse. However, human TFs regulate larger number of different clusters, than mouse TFs. It should also be noted that none of undifferentiated ESC-related TFs (OCT4, SOX2, and NANOG) is found to contribute to the regulation: all these are uniformly down-regulated during the development process.

## Discussion

We performed a comparison of gene expression in hESCs and mESCs during their early differentiation. Different microarray platforms are used to quantify the expression in these two cases, making direct comparison not straightforward, although a high degree of correlation between gene expression levels in human and mouse cells is anticipated, as well as seen previously, when the measurements were directly comparable [13]. In this work, we found that selection of the probe sets of orthologous genes, which have the largest variation in their signals, together with normalization allows establishing a correspondence between gene expression time courses in two species. We find that a majority of differentially regulated orthologous genes have correlated expression changes.

This correspondence we use further to define gross similarities and differences in the development of differentiation. These are quantified for about 2500 genes found to be significantly differentially expressed in both hESCs and mESCs. When expression profiles are grouped by means of clustering methods, majority of clusters show similar expression. There is a range of divergent patterns during the development process. The largest pattern, which is the same in both ESCs, is slow down-regulation of several hundred genes related to cell cycle (as well as RNA processing, DNA metabolic process). There is a sharp down-regulation of several hundred genes within the first days of development in both mESCs and hESCs, which is not conserved, i.e. the down-regulation takes place in both cases, but the genes with this pattern are not the same. We could identify these genes either related to the “response to stimulus” or “transcriptional regulation”, which are ESC type specific. Both ESCs have peak-wise regulation of specific groups of genes, related to embryonic morphogenesis, but the positions of peaks in time is not the same, and as such it is difficult to pick out any conservation. One can state that neuron development processes in hESCs occur much later than in mESCs. There are other quantifiable differences for other development processes.

Interestingly, the most appropriate time scale matching was roughly matching 10 days of mESC development to 21 days of hESC development. This probably may be attributed to differences in population doubling time 12–15 h for mESCs *vs* 30–35 h for hESCs [15]. Apart from this there is a difference in the number of upregulated and expressed during the development process genes. Namely, one can state that mESCs have detectably more genes involved in the development process. This fact may contribute to even faster development of ESCs in mouse.

Differences in gene expression patterns are expected to be related to differences in regulatory mechanisms. Differences in regulation of orthologous genes may be due to complex reasons, for example, Xie et al. [12] found that two classes of genomic changes may contribute to interspecies expression difference: single nucleotide mutations leading to turnover of transcription factor binding sites, and insertion of cis-regulatory modules (CRMs) by transposons. We quantified the regulation mechanisms by lists of TFBSs on the promoters of gene groups. Comparing human and mouse gene groups, we find that similar expression patterns are as a rule accompanied with similar sets of assigned TFBSs. Surprisingly, there was a case in which slow down-regulation in both ESCs could not be attributed to common TFBSs, indicating that the mechanisms other than transcription factor binding may be in action there. In a few cases, expression patterns differ significantly albeit the TFBSs on the promoters are conserved, indicating species-dependent activity of transcription factors regulating these genes. We also found groups of genes with both different expression patterns as well as apparently different promoters, most likely corresponding to the case of orthologous genes having nonconserved promoters.

## Materials and Methods

### Cell preparation and microarray data

Microarray data for human ESCs, measured by [10], and for mouse, measured by [11] were used in our analyses. H9 hESCs (WiCell, Madison, WI, USA) were cultured in DMEM-F12, 20% KO serum replacement, 1% non-essential amino acids, penicillin (100 units/ml), streptomycin (100 μg/ml), and 0.1 mM β-mercaptoethanol supplemented with 4 ng/ml basic fibroblast growth factor (bFGF). The cells were passaged with mechanical dissociation on irradiated mouse embryonic fibroblasts (MEF). Prior to differentiation, the cells were maintained for five days in 60-mm tissue culture plates (Nunc, Langenselbold, Germany) coated with a hESC-qualified matrix (BD Biosciences, California, USA) in mTESR medium (Stem Cell Technologies). For the time-kinetic multilineage differentiation, embryoid bodies (EBs) were prepared with minor changes (60 to 70 clumps were added instead of 50 to 60), and the EBs were maintained for 21 days on a horizontal shaker. All of the experiments were performed as three independent (n = 3) biological replicates.

CGR8 ESCs (ECACC 95011018) were cultured without feeder cells in Glasgow minimum essential medium supplemented with 10% fetal bovine serum (FBS), 2 mM l-glutamine, 100 U/mL LIF, and 50μM β-mercaptoethanol (ME) in 0.2% gelatine-coated flasks. CGR8 ESCs (passage No. 8) were treated with trypsin and used for preparation of cell suspensions (25,000 cells/mL) in Iscove's modified Dulbecco's minimal essential medium (IMDM) (Invitrogen) supplemented with 20% FBS, 1% non-essential amino acid (NEAA) (vol/vol), 2 mM l-glutamine, and 100μM β-ME. For the “hanging drop” method, 20μL drops of this ESC suspension were placed on the inner surface of the lid of a Petri dish (diameter: 10 cm; Greiner). The Petri dishes that contained 5 mL phosphate-buffered saline (PBS) were closed with the lid and incubated under normal culture conditions. After days 1–7 and 10, all EBs were separately collected for RNA isolation. RNA isolation from the 10-day-old EBs was performed after the 7-day-old EBs were plated in cultured dishes and incubated in IMDM supplemented with 20% FBS, 100μM β-ME, 2 mM l-glutamine, and 1% NEAA (vol/vol) for 3 more days. Total RNAs from these time points were taken for the transcriptome study.

### Data analysis and statistical procedures

Background correction and normalization were performed with R Bioconductor package affy. We used RMA method [24] to calculate expression values for each microarray and MAS 5.0 Presence/Absence calls to obtain Presence/Absence calls and their associated Wilcoxon p-values. Various scripts were used to extract gene lists with significant changes in expression values, differences in expression profiles and presence calls at various conditions. We applied Fisher transformation [25] of sample correlation coefficients to derive statistically significant differences in expression profiles of human and mouse genes. DAVID annotation service [26] was used to classify gene groups with discriminative GO Biological Process classification.

For clustering, we selected orthologous gene pairs, which were differentially regulated in both hESCs and mESCs. Aiming at 2500 genes for the analysis we selected half of the probe sets with log2 fold change larger than 0.5 with respect to the average expression level in both mouse and human data. Selecting the most varying probe set for each gene and overlapping human and mouse gene lists according to orthology relation resulted in 2587 gene pairs. Clustering was done with CRC [27].

We tested the reproducibility of results by repeating the analysis using mean expression levels instead of selected-probe (having the largest variation) levels. The differences were minor. The reason for this is overall similarity of averaged *vs* single-probe expression profiles for the same gene in our data sets. Among selected 2587 genes, 2530 (97.8%) selected-probe profiles were significantly (Pearson Correlation Coefficient, PCC > 0.67, see the next paragraph) correlated with respective mean profiles in mouse dataset, in spite of average number of probes per gene 2.58. In case of human dataset, 2289 (88.1%) selected-probe profiles were correlated (PCC > 0.71) with respective mean profiles, at the average number of probes per gene 2.95.

One can use several methods to calculate and rate similarity of profiles from replicated measurements, e.g., comparing averaged profiles, or averaging 3x3 correlation coefficients, transforming calculated 3x3 Pearson coefficients to normally distributed variables and deriving correlation coefficients at a given p-value. We adopted PCCs calculated using averaged profiles and assigned them statistical significance dependent on the number of points (NP) in time series measurements. Namely, PCC was converted to t-test statistics

$$t=PCC\times \sqrt{NP-2}/\sqrt{1-{PCC}^{2}}$$

which was then used to calculate p-value via two-tailed test. P-value is less than 0.05, when PCC>0.71 for NP = 8 hESC time series data, and PCC>0.67 for NP = 9 mESC data. F-Match program [21] was used to find TFBSs on the promoters of selected gene groups. As an additional condition, it was required that at least 40% of promoters of the genes in the group should have at least one TFBS. Gene groups for these computations were selected as the best 50 representatives of clusters. As “background”, we used 500 genes found to be expressed according to Presence/Absence calls and located at the bottom of the list of genes, sorted by the magnitude of expression level variation (the probe set with the largest variation was presenting the gene). We also applied GC content correction to the background promoters, as it was found to be resulting in different predictions for TFBS-s [28, 29]. The conclusions about the differences and similarity of promoters did not change after this correction.

## Supporting Information

S1 Table Table of Presence/Absence calls for all gene pairs. Number of Presence/Absence calls in all arrays for 15073 orthologous gene pairs in human and mouse.
(XLSX)

S2 Table Table of Presence/Absence calls for TF pairs. Number of Presence/Absence calls in all arrays for 1072 orthologous transcription factor pairs in human and mouse.
(XLSX)

S3 Table Table of human/mouse gene pairs with FC and correlation of expression. For selected differentially regulated 2587 gene pairs, maximal fold change in human and mouse arrays, correlation of expression profiles in human and mouseare presented.
(XLSX)

S4 Table Table with the clusters derived from hESC arrays. Clusters are described by the number of genes in them, top 5 enriched GO Biological Processes and average expression profile in hESCs and mESCs.
(PDF)

S5 Table hESC clusters presented by probe sets. Probeset IDs for genes of the clusters from S4 Table.
(XLSX)

S6 Table hESC clusters with gene description. Annotation of genes of the clusters from S4 Table.
(TXT)

S7 Table Table with the clusters derived from mESC arrays. Clusters are described by the number of genes in them; top 5 enriched GO Biological Processes and average expression profile in hESCs and mESCs.
(PDF)

S8 Table mESC clusters presented by probe sets. Probeset IDs for genes of the clusters from S7 Table.
(XLSX)

S9 Table mESC clusters with gene description. Annotation of genes of the clusters from S7 Table.
(TXT)

S10 Table Lists of relevant TFs for hESC gene groups. TFs with binding sites found to be overrepresented with p-value <10^−9^ on the promoters of clusters.
(XLSX)
