# Supplementary material for: Differences in the Early Development of Human and Mouse Embryonic Stem Cells
Source: PLoS One. 2015 Oct 16;10(10):e0140803. doi: 10.1371/journal.pone.0140803 (PMC4608779; doi:10.1371/journal.pone.0140803)
Supplement: S4 Table — Clusters are described by the number of genes in them, top 5 enriched GO Biological Processes and average expression profile in hESCs and mESCs. (PDF) [file pone.0140803.s004.pdf]

Human Clusters

NCluster, NGenes
GOTERM_BP

Espression HS, MU

P

0001, 268
M phase 30 3E-14
DNA replication 19 8E-10
nuclear division 20 1E-9
cell cycle 36 9E-9
cell division 19 7E-7

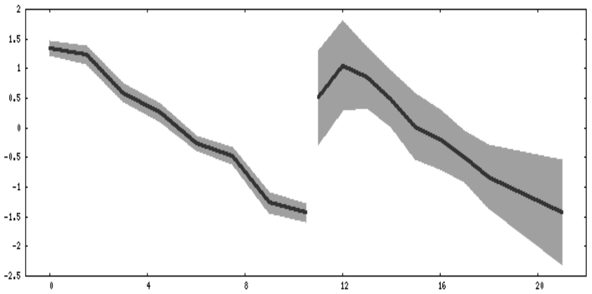

-

0002, 213
mitochondrion organization 8 0.002
death 19 0.003
response to hypoxia 7 0.006
iron ion homeostasis 4 0.01
androgen receptor signaling pathway 4 0.01

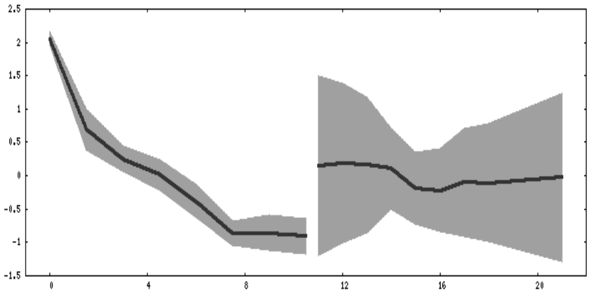

-

0003, 198
cell projection morphogenesis 10 7E-4
cell part morphogenesis 10 0.001
neuron differentiation 13 0.001
positive regulation of developmental process 10 0.002
neuron development 11 0.002

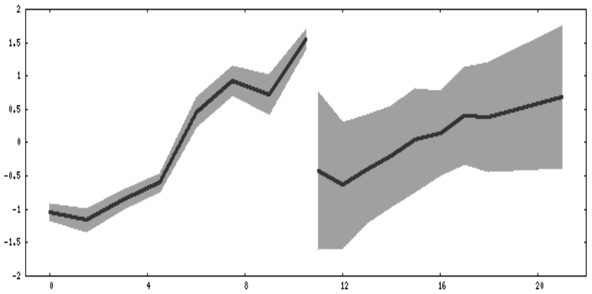

+

0004, 166
regulation of muscle cell differentiation 7 1E-6
embryonic morphogenesis 14 4E-6
regulation of transcription 44 3E-5
pattern specification process 12 3E-5
regulation of cell proliferation 20 9E-5

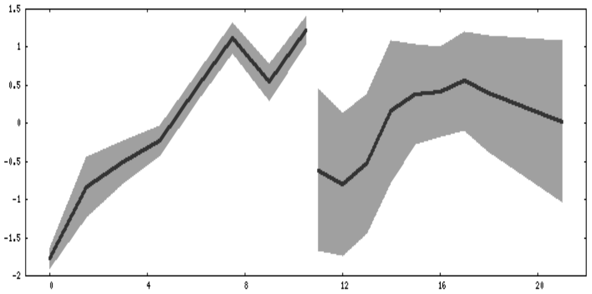

+

0005, 158
regulation of transcription 42 3E-5
negative regulation of macromolecule biosynthetic process 16 7E-5
chromatin modification 9 0.002
vasculature development 8 0.006
skeletal system development 9 0.006

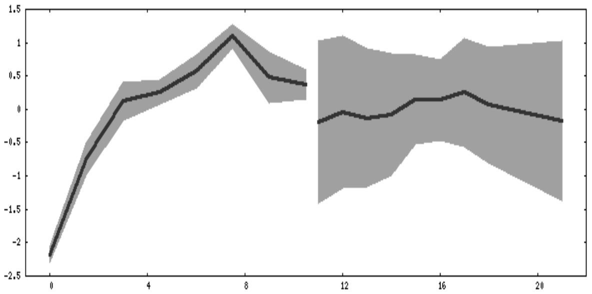

+

0006, 153
regulation of cell-substrate adhesion 6 5E-5
response to endogenous stimulus 14 7E-5
response to organic substance 19 7E-5
intracellular signaling cascade 25 3E-4
interphase of mitotic cell cycle 7 3E-4

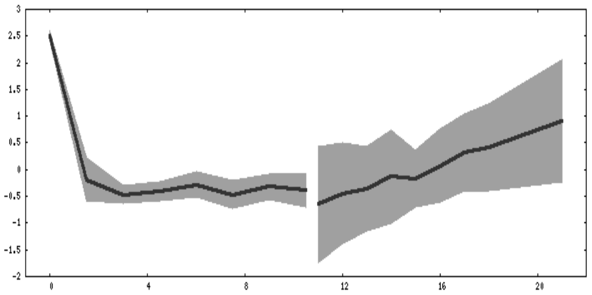

+

0007, 132
extracellular matrix organization 13 3E-11
skeletal system development 13 9E-6
blood vessel development 11 3E-5
vasculature development 11 3E-5
bone development 7 4E-4

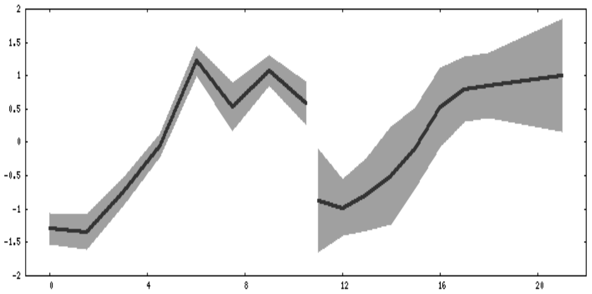

?

0008, 129
neuron development 12 2E-5
cell morphogenesis 11 1E-4
cell motion 9 0.02
regulation of metal ion transport 4 0.02
growth 5 0.04

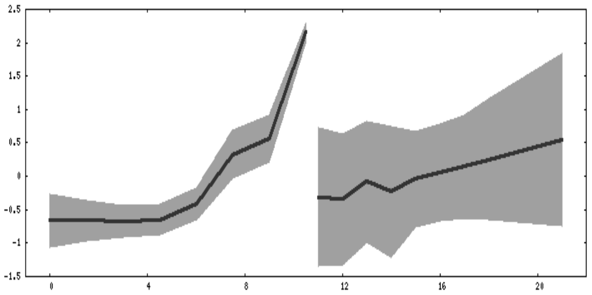

+

0009, 106
mRNA processing 10 2E-4
RNA splicing 9 4E-4
tube development 8 4E-4
kidney development 5 0.003
regulation of transcription 27 0.007

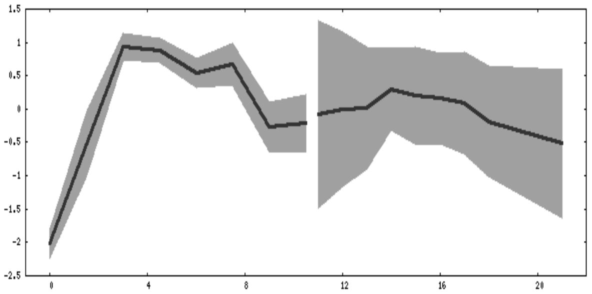

~

0010, 101
negative regulation of transcription,
DNA-dependent 10 2E-4
DNA metabolic process 11 8E-4
ncRNA processing 7 8E-4
transcription 24 0.002
DNA replication initiation 3 0.004

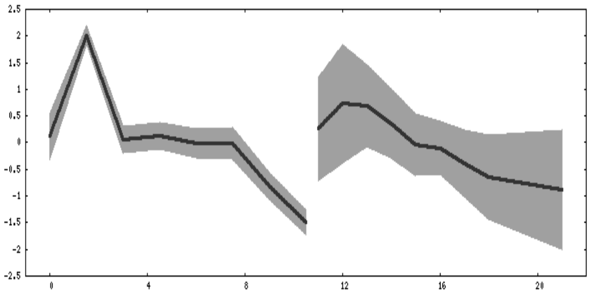

+

0011, 89
negative regulation of macromolecule
metabolic process 14 2E-4
apoptotic mitochondrial changes 4 6E-4
negative regulation of gene expression 10
0.002
cellular response to extracellular stimulus 4
0.005
regulation of translation 5 0.007

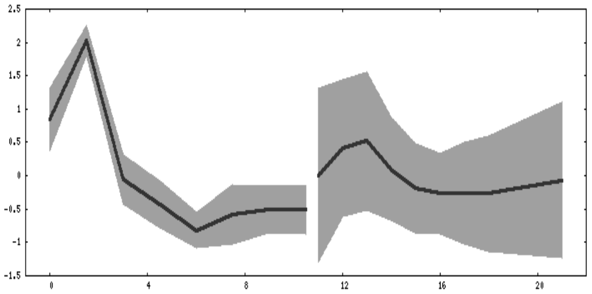

-

0012, 84
cell cycle phase 12 4E-6
nuclear division 7 6E-4
organelle fission 7 8E-4
blastocyst development 4 0.001
stem cell maintenance 3 0.006

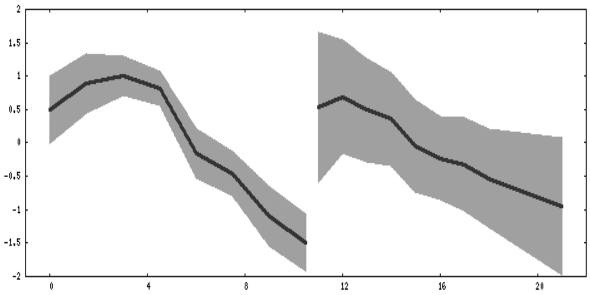

~

0013, 76
cell adhesion 8 0.03
carbohydrate transport 3 0.03
response to abiotic stimulus 5 0.07
intracellular signaling cascade 10 0.08
regulation of transcription from RNA polymerase II promoter 7 0.08

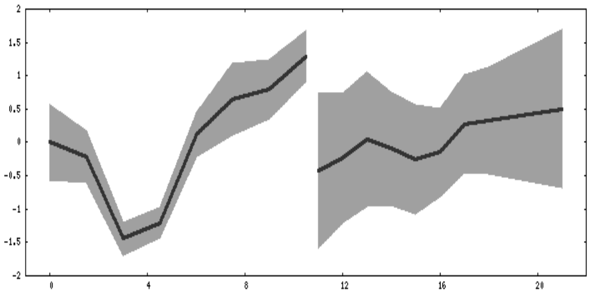

+

0014, 74
regulation of transcription 23 0.002
cell cycle 10 0.008
embryonic morphogenesis 6 0.01
cellular response to stress 8 0.01
response to DNA damage stimulus 6 0.03

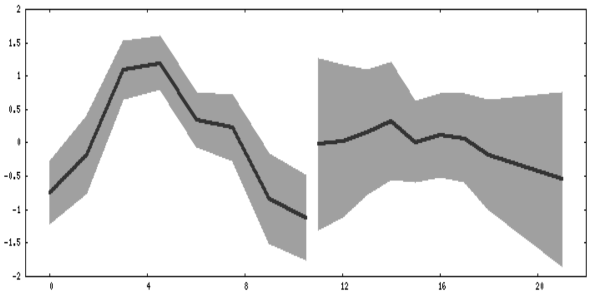

+

0015, 71
response to organic substance 11 5E-4
tube development 5 0.01
cell growth 3 0.02
positive regulation of developmental process 5 0.02
regulation of apoptosis 8 0.04

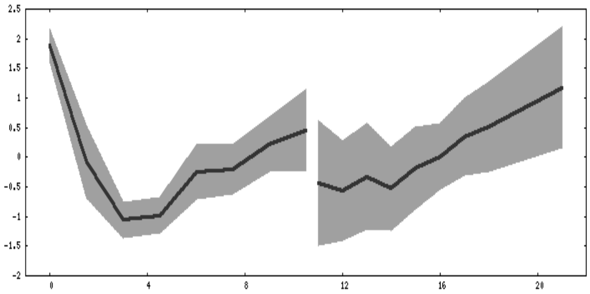

+

0016, 71
embryonic morphogenesis 13 5E-9
tube development 10 4E-7
mesoderm development 6 2E-5
limb morphogenesis 6 6E-5
heart development 7 3E-4

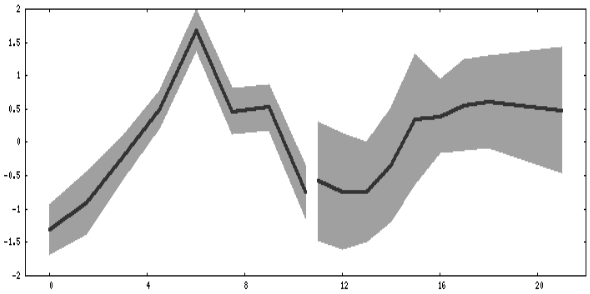

+

0017, 70
L-serine metabolic process 3 5E-4
cell adhesion 10 0.002
negative regulation of apoptosis 7 0.004
muscle organ development 5 0.01
heart development 5 0.01

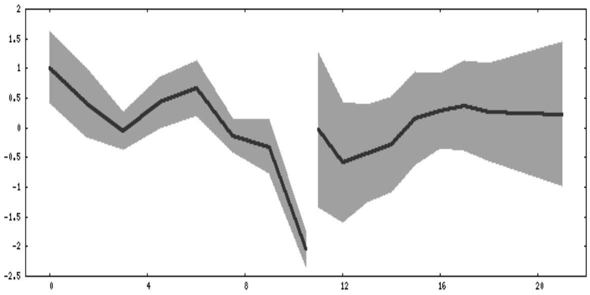

+

0018, 61
response to endogenous stimulus 7 0.002
response to metal ion 4 0.009
~response to hypoxia 4 0.01
regulation of cell proliferation 7 0.04
muscle tissue development 3 0.06

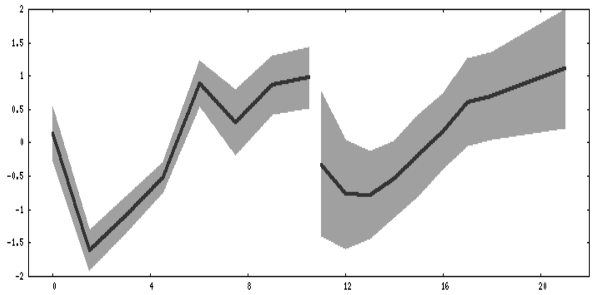

+

0019, 46
embryonic morphogenesis 6 7E-4
cell motion 7 8E-4
embryonic organ development 4 0.008
regulation of nervous system development 4 0.01
blood vessel morphogenesis 4 0.01

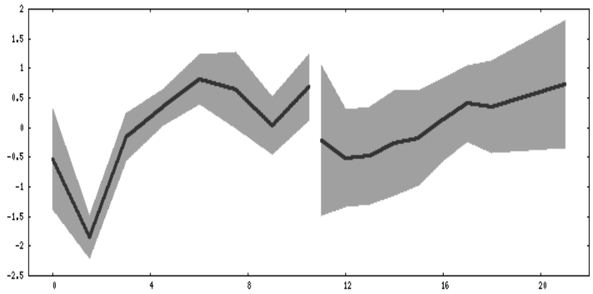

-

0020, 45
transcription 15 4E-4
tissue morphogenesis 4 0.01
tube development 4 0.02
epithelium development 4 0.02
lung development 3 0.03

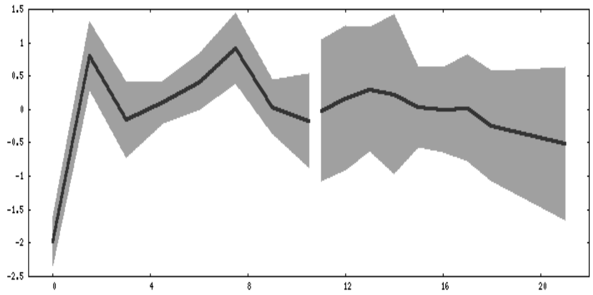

~

0021, 27
hexose metabolic process 4 0.003
response to hypoxia 3 0.02
proximal/distal pattern formation 2 0.04
negative regulation of gene expression 4 0.04
skeletal system development 3 0.09

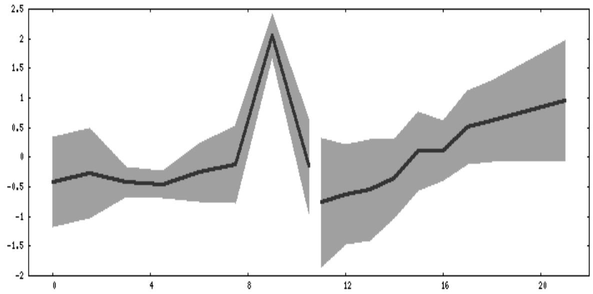

-

0022, 26
regulation of cell motion 4 0.002
phosphorylation 5 0.02
phosphate metabolic process 5 0.04
cell adhesion 4 0.06
protein kinase cascade 3 0.09

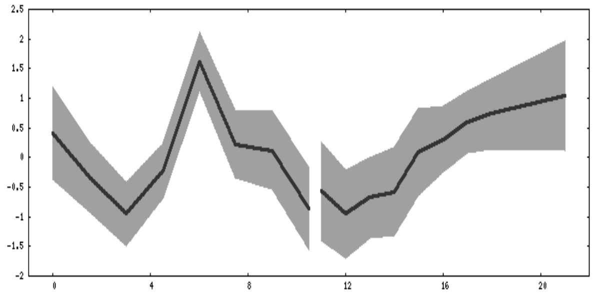

-

0023, 9

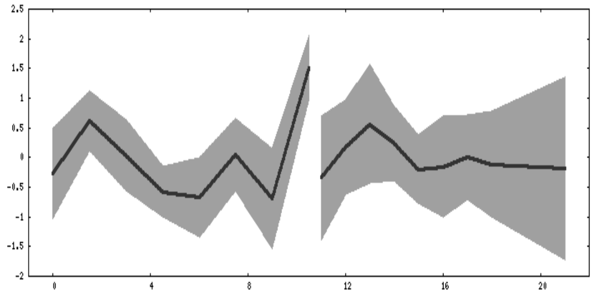

+
