# Supplementary material for: Differences in the Early Development of Human and Mouse Embryonic Stem Cells
Source: PLoS One. 2015 Oct 16;10(10):e0140803. doi: 10.1371/journal.pone.0140803 (PMC4608779; doi:10.1371/journal.pone.0140803)
Supplement: S7 Table — Clusters are described by the number of genes in them; top 5 enriched GO Biological Processes and average expression profile in hESCs and mESCs. (PDF) [file pone.0140803.s007.pdf]

Mouse clusters

N, NGene
GOTERM_BP

Expression HS, MU

P

0001, 260
RNA processing 39 3E-15
DNA metabolic process 33 8E-12
cell cycle 36 8E-9
DNA replication 17 4E-8
RNA splicing 20 8E-8

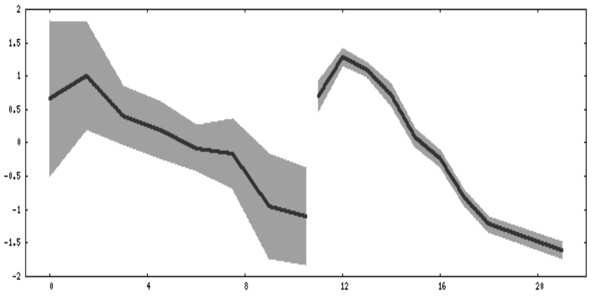

-

0002, 221
tissue morphogenesis 12 9E-6
skeletal system development 15 3E-5
vasculature development 12 2E-4
heart development 11 2E-4
tube development 10 0.001

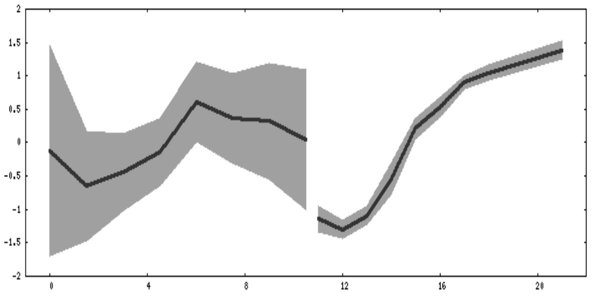

+

0003, 183
regulation of transcription 43 0.002
regulation of apoptosis 19 0.002
serine family amino acid biosynthetic process 3 0.006
amine biosynthetic process 5 0.01
liver development 4 0.02

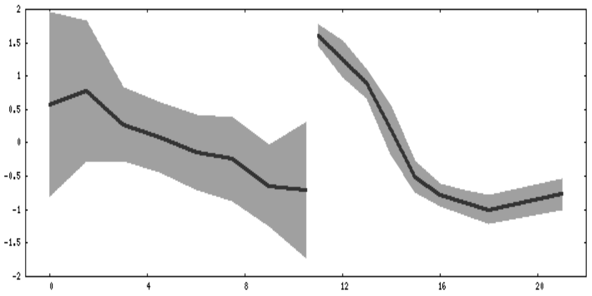

?

0004, 174
regulation of transcription from RNAPII prom 19 2E-4
cell death 18 5E-4
vasculature development 10 7E-4
heart development 9 0.001
tube development 9 0.001

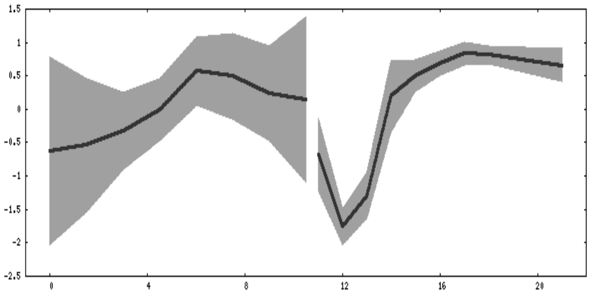

+

0005, 150
extracellular structure organization 11 1E-6
vasculature development 11 6E-5
muscle organ development 10 9E-5
skeletal system development 12 1E-4
blood vessel development 10 3E-4

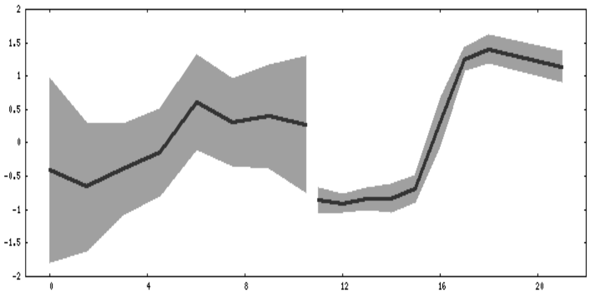

+

0006, 118
cell cycle 38 1E-22
cell division 24 1E-18
DNA metabolic process 23 2E-12
response to DNA damage stimulus 17 3E-9
chromosome segregation 10 3E-9

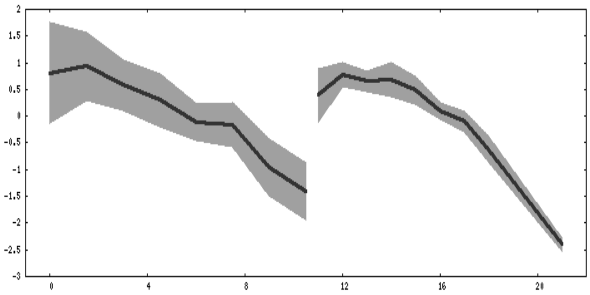

+

0007, 115
heart development 10 1E-5
tube development 10 2E-5
embryonic morphogenesis 10 2E-4
blood vessel development 9 2E-4
vasculature development 9 3E-4

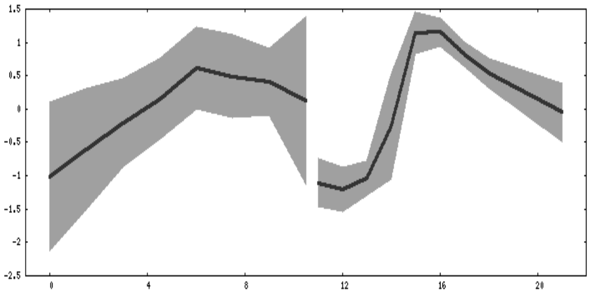

+

0008, 111
neuron development 10 3E-4
cell projection organization 9 0.002
regeneration 4 0.01
cell morphogenesis involved in differentiation 6 0.02
protein homooligomerization 4 0.02

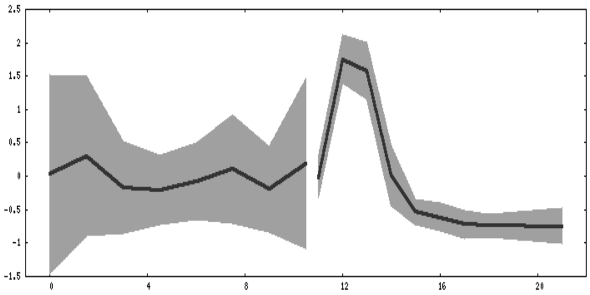

+

0009, 109
response to organic substance 17 3E-5
anti-apoptosis 9 8E-5
muscle organ development 9 1E-4
regulation of apoptosis 16 4E-4
response to endogenous stimulus 11 5E-4

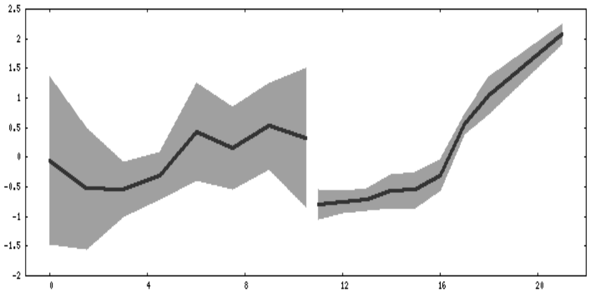

+

0010, 105
positive regulation of macromolecule metabolic process 15 3E-4
regulation of transcription from RNA polymerase II promoter 13 9E-4
positive regulation of gene expression 11 0.002
positive regulation of biosynthetic process 12 0.002
negative regulation of macromolecule metabolic process 12 0.003

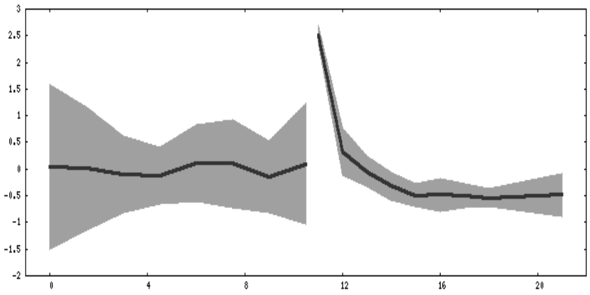

-

0011, 105
regulation of intracellular transport 6 7E-5
tissue morphogenesis 7 8E-4
regulation of cell development 7 0.002
tube morphogenesis 5 0.008
anti-apoptosis 6 0.009

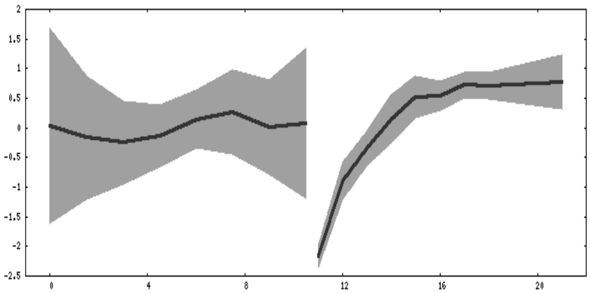

+

0012, 89
neuron development 8 0.002
regulation of ion transmembrane transporter activity 3 0.007
cell morphogenesis involved in differentiation 6 0.008
cell morphogenesis 7 0.009
cell projection organization 7 0.01

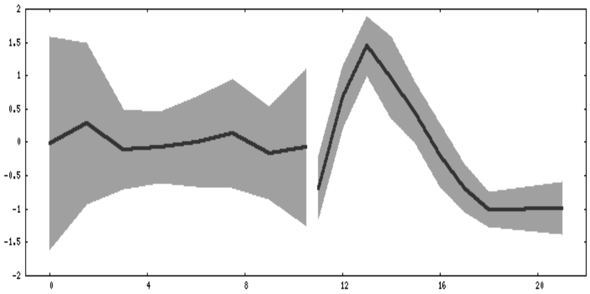

+

0013, 81
negative regulation of phosphorylation 5 4E-5
regulation of cell proliferation 13 1E-4
defense response 10 0.001
response to inorganic substance 6 0.002
regulation of cell cycle 7 0.003

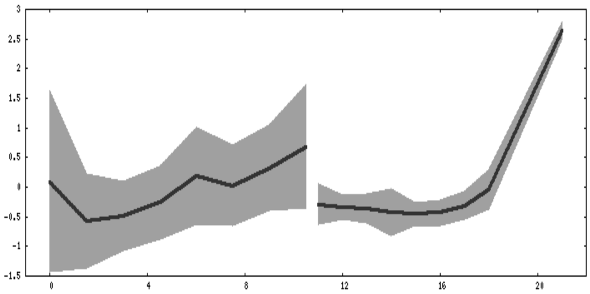

+

0014, 72
response to oxidative stress 5 0.004
response to extracellular stimulus 5 0.01
anti-apoptosis 4 0.04
ear development 3 0.05
cell proliferation 5 0.09

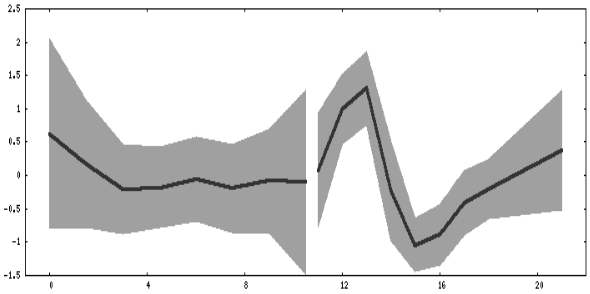

+

0015, 70
response to organic substance 11 6E-4
extracellular matrix organization 5 8E-4
regulation of cell growth 6 0.001
phospholipid transport 3 0.008
monosaccharide biosynthetic process 3 0.01

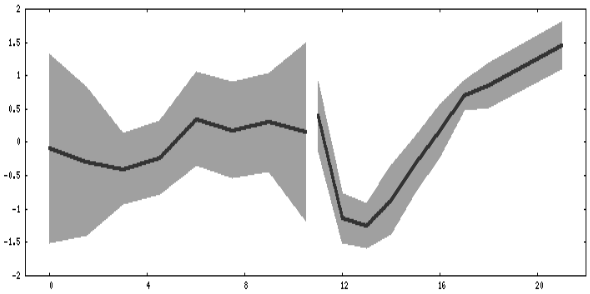

+

0016, 69
enzyme linked receptor protein signaling pathway 8 2E-4
embryonic limb morphogenesis 4 0.004
embryonic morphogenesis 6 0.005
negative regulation of molecular function 6 0.008
cell growth 3 0.02

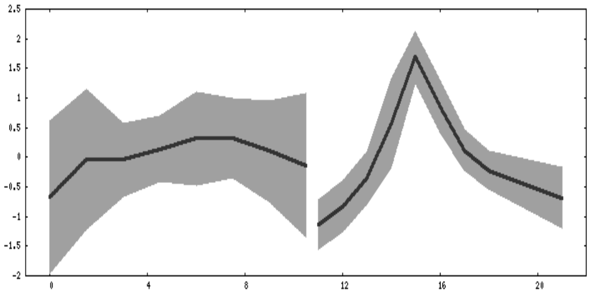

+

0017, 65
lung development 4 0.007
lipid catabolic process 4 0.03
negative regulation of gene expression 6 0.05
tube development 4 0.06
monosaccharide metabolic process 4 0.06

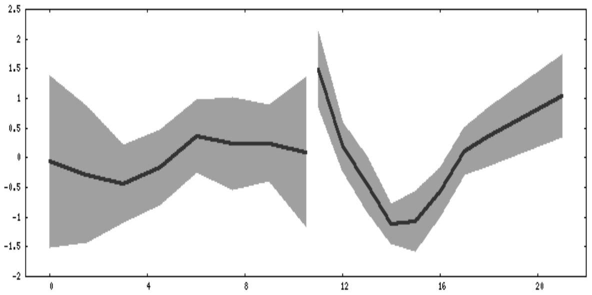

+

0018, 65
cell adhesion 10 6E-4
blood vessel development 4 0.05
hemostasis 3 0.05
vasculature development 4 0.06
proteolysis 8 0.07

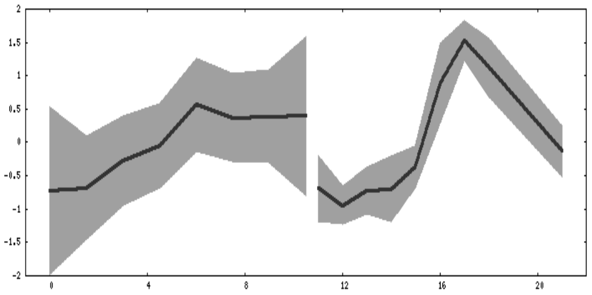

+

0019, 61
pattern specification process 12 5E-9
embryonic morphogenesis 12 2E-8
forebrain development 7 3E-5
tube development 7 2E-4
sensory organ development 7 3E-4

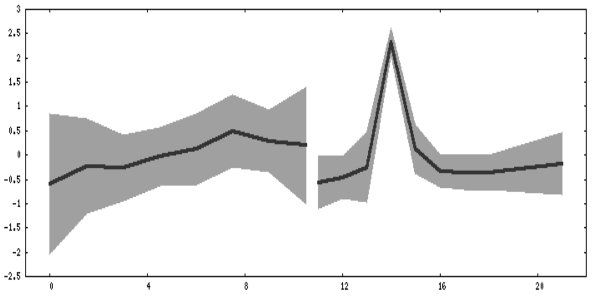

+

0020, 60
sterol biosynthetic process 4 3E-4
regulation of RNA metabolic process 14 0.01
lipid biosynthetic process 5 0.03
negative regulation of gene expression 6 0.04
posttranscriptional regulation of gene expression 4 0.04

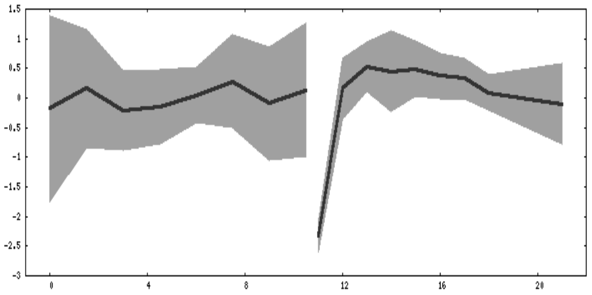

+

0021, 50
regulation of skeletal muscle fiber development 3 0.002
regulation of muscle development 3 0.007
regulation of cell motion 3 0.08
regulation of cell development 3 0.09
cell morphogenesis inv. neuron differentiation 3 0.09

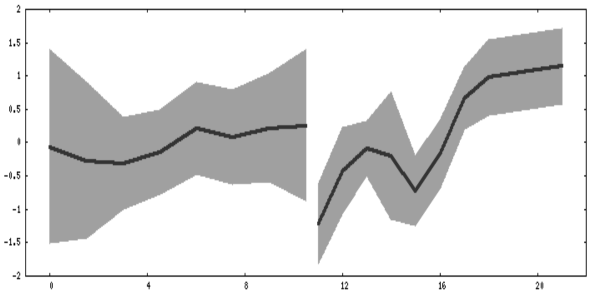

+

0022, 48
M phase 6 0.002
cell cycle phase 6 0.005
DNA packaging 3 0.04
cell division 4 0.05
limbic system development 2 0.08

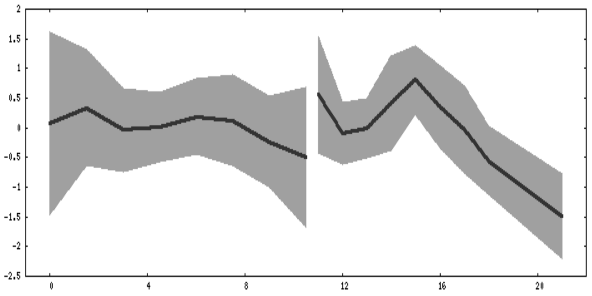

-

0023, 36
response to abiotic stimulus 5 0.007
response to hypoxia 3 0.03
glucose metabolic process 3 0.04
hexose metabolic process 3 0.06
fatty acid metabolic process 3 0.06

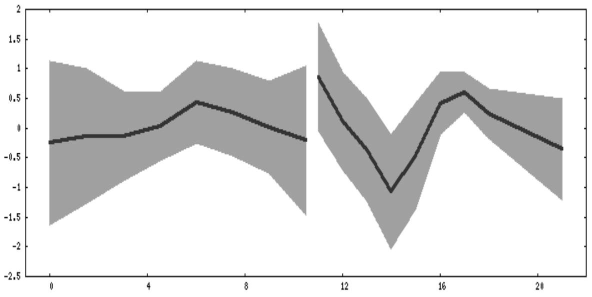

+
